# Chemistry behind Quality—Emission of Volatile Enantiomers from *Mentha* spp. Plant Tissue in Relationship to Odor Sensory Quality

**DOI:** 10.3390/foods12102057

**Published:** 2023-05-19

**Authors:** Jacek Łyczko, Anna Kiełtyka-Dadasiewicz, Hanán Issa-Issa, Mariusz Skrzyński, Renata Galek, Ángel A. Carbonell-Barrachina, Antoni Szumny

**Affiliations:** 1 Department of Food Chemistry and Biocatalysis, Wrocław University of Environmental and Life Sciences, 50-375 Wrocław, Poland; antoni.szumny@upwr.edu.pl; 2 Department of Plant Production Technology and Commodity Science, University of Life Sciences in Lublin, 20-950 Lublin, Poland; anna.kieltyka-dadasiewicz@up.lublin.pl; 3 Garden of Cosmetic Plants and Raw Materials, Research and Science Innovation Center, 20-819 Lublin, Poland; 4 Research Group ‘Food Quality and Safety’, Department of Agro-Food Technology, Escuela Politécnica Superior de Orihuela, Universidad Miguel Hernández de Elche (UMH), 03312 Orihuela, Spain; hissa@umh.es (H.I.-I.); angel.carbonell@umh.es (Á.A.C.-B.); 5 Department of Plant Breeding and Seed Production, Wrocław University of Environmental and Life Sciences, 50-363 Wrocław, Poland; mariusz.skrzynski@upwr.edu.pl (M.S.); renata.galek@upwr.edu.pl (R.G.)

**Keywords:** VOCs, mint, gas chromatography mass spectrometry, medicinal and aromatic plants, HS-SPME, enantiomeric distribution

## Abstract

The quality of food, considering increasing consumer demands and competition among producers, is a highly important issue. Quality concerns are also applicable to the odor quality of herbs and spices (HSs). Meanwhile, HSs commonly are graded based on their essential oils (EOs) content and analysis; but does the instrumental analysis really provide general information about the HSs sensory quality? Three chemotypes of *Mentha* spp. were used in the present study. From samples diversified by convective drying at different temperatures, EOs were hydrodistillated and analyzed by enantioselective GC-MS; moreover, the source plant material’s volatile profile was analyzed by the HS-SPME technique. The instrumental analysis was confronted with the results of the sensory panel. Changes in enantiomeric composition were observed during the drying process, although no clear correlations or trends could be found for individual chiral components. Furthermore, even with significant differences in particular volatiles’ contribution to plants’ EOs and their volatile profiles, judges were not able to match the sample EOs and plant samples with sufficient effectiveness (~40%). Based on those results, we suggest that volatile enantiomeric distribution does not have an actual influence on odor quality and that the sensory analysis should not be replaced with instrumental analysis, which cannot predict general sensory quality.

## 1. Introduction

Medicinal and aromatic plants (**MAPs**) are a large group of plant families (e.g., *Lamiacae, Astereace*), which have a considerable place in the pharmaceutical, cosmetics, and food industries [1]. Among MAPs, a special place is held for herbs and spices (**HSs**), which despite their properties beneficial for health, possess unique flavoring features [2]. Due to those flavoring features, the quality assessment of HSs has a crucial role for companies during raw-material purchases, for technologists during processing, and finally, for consumers during usage.

The flavoring features of HSs are mainly caused by significant amounts of essential oils (**EOs**) present in the plant tissue, which are responsible for the characteristic aroma of HSs [3]. In general, the EOs yield was considered the most important factor for the determination of HSs sensory quality and origin [4,5], however, actual studies prove that this factor was overrated [6,7,8]. The very first stage of this perspective change was the determination that more relevant than the EOs yield is its composition and mutual volatile organic compounds (**VOCs**) ratio [8]. Moreover, the latest data show that not only the VOCs ratio should be considered, but also the influence of plant tissue presence, which limits the emission of VOCs composing plant EOs [6]. The further steps may consider a more in-depth investigation of the enantiomeric distribution of HSs volatiles in the plant’s headspace.

The most measurable method for HSs sensory quality determination is its assessment by a trained panel. Sensory analysis is nonetheless time-consuming and expensive, due to the need for panel judges’ training and senses calibration each time when specific material is evaluated. Therefore, the seeking of trends between sensory quality evaluation and chemical analyses is highly in demand. For this reason, the study focused on looking for the connection between instrumental and sensory analysis, considering the VOCs enantiomeric composition design. The chemical analysis of the enantiomeric composition of EOs by GC-MS and the emission of VOCs enantiomers to plant headspace by HS-SPME was confronted with the results of sensory quality assessment of whole plants and obtained from the EOs. The study was performed on the example of three *Mentha* spp. chemotypes (carvone pathway, menthol pathway, and linalool pathway). The *Mentha* spp. plants were chosen as an object of the present research because of their wide availability in Poland, which ensured the required amount of material for experiment purposes. Furthermore, several *Mentha* spp. plants were identified in previous research as those abundant in enantiomeric VOCs [5,9].

## 2. Materials and Methods

### 2.1. Chemicals

For chemical analyses, EOs were diluted in cyclohexane MS SupraSolv^®^ for gas chromatography (Sigma-Aldrich, Steinheim, Germany). For enantiomers identification, the analytical standards of (−)-menthol, (+)-menthol, (−)-menthone, (+)-menthone, (−)-α-terpineol, (+)-α-terpineol, (−)-limonene, (+)-limonene, (−)-terpinen-4-ol, (+)-terpinen-4-ol, (−)-*trans*-caryophyllene, (−)-carvone, (+)-carvone, (−)-linalool, (±)-linalool, (−)-menthyl acetate, (+)-menthyl acetate, (−)-dihydrocarvone, (+)-dihydrocarvone, (−)-borneol, and undecane-2-one (all Sigma-Aldrich, Steinheim, Germany) were used.

### 2.2. Plant Material

Mint samples of cultivars were obtained from the Garden of Cosmetic Plants and Raw Materials, Research and Science Innovation Centre (Lublin, Poland) and cultivated at Wrocław University of Environmental and Life Sciences research station Swojczyce (Wrocław, Poland). The field was classified as arable soils of average quality, better soil (Class IVa according to the Polish bonitation class) that was slightly acidic (pH 6.3). The harvesting was performed in July 2021, when the plants reached the flower bud phase (BBCH 54). Mint cultivars are classified according to particular chemotypes due to the presence of specific enzymes in the plant’s metabolic pathways. For all mint samples, the biosynthesis of characteristic VOCs (menthol, carvone, or linalool) is related to the mevalonate pathway. The starting point of all three chemotypes is geranyl PP followed by linalyl PP. From this step, the pathway branches out in two ways: linalool and limonene. Then, limonene as an intermediate may be converted into pulegone and further reduced to menthol. In addition, limonene branches out the pathway to carvone via conversion to carveole.

After the harvesting, the plant material within the cultivars was precisely mixed and subjected to different post-harvest treatments: vacuum packaging and storing at −18 °C as reference material; convective drying at 40 °C; convective drying at 55 °C; convective drying at 70 °C. The drying process was introduced into the experiment design to differentiate the material to increase the objects for trends observation. The list of samples is presented in Table 1.

### 2.3. EOs Hydrodistillation and Sample Preparation

Hydrodistillation of EOs was conducted with the Deryng-type apparatus. Briefly, 100 ± 0.05 g of shredded frozen material or 10 ± 0.05 g of dried material was placed in a round bottom flask. Then, the material was suspended in 250 mL or 150 mL of distilled water, respectively. The distillation was performed for 45 min after the start of boiling. After the process, the EOs were collected and measured with 0.05 mL accuracy. The EOs yield was calculated as % of dry weight [*v*/*w*]. The samples were stored at −18 °C before analysis. All samples were distilled with three replications.

For GC-MS analysis, 5 µL of EOs with 25 µg of undecane-2-one as internal standard was diluted up to 1 mL with cyclohexane and placed into a 1.5 mL chromatographical vial. Samples obtained by drying at 70 °C, due to significantly lower amounts of EOs obtained, were not included in further chemical analyses.

### 2.4. HS-SPME Arrow Extraction

Extraction of VOCs was performed with 1.10 mm DVB/C-WR/PDMS SPME Arrow fiber (Shimadzu, Kyoto, Japan). Briefly, 1 ± 0.005 g of frozen or 0.1 ± 0.005 g of dried material was used for VOCs extraction from plant material. The extraction was performed in 20 mL headspace vials for 30 min at 45 °C with the addition of 25 µg of undecane-2-one as the internal standard. The extraction proceeded with incubation for 10 min at the same temperature. The analytes were desorbed at the conditions of the GC injection port for 3 min.

### 2.5. GC-MS Analysis

Enantiomeric analysis of mint EOs and VOCs was performed with Shimadzu GCMS QP 2020 Plus (Shimadzu, Kyoto, Japan) equipped with a Cydex-B capillary column (50 m × 0.25 mm × 0.25 µm; Trajan Scientific Europe Ltd., Milton Keynes, UK). GC operational conditions were as follows: injection port 220 °C; temperature program started with 60 °C held for 2 min, then at the of rate 3 °C·min^−1^ to 150 °C, then at the rate of 25 °C·min^−1^ to 220 °C; helium as carrier gas with flow 0.9 mL·min^−1^; split 75 (liquid injection) or 100 (HS-SPME Arrow analysis). MS operational conditions were as follows: interface temperature 210 °C; ion source temperature 250 °C; scan 35–350 *m/z*.

The identification of enantiomers was performed by simultaneous analysis of the analytical standards and in reference to Alvarez-Rosas et al. (2022) [10] study. The quantification was based on the peak area normalization performed against the internal standard peak area.

### 2.6. Sensory Analysis

Ten trained panelists from the Food Quality and Safety Group (Escuela Politécnica Superior de Orihuela) of the Universidad Miguel Hernández de Elche (Orihuela, Alicante, Spain), selected according to ISO standard 8586-1 [11,12], performed the sensory analyses. Before the actual panel, the panelists were trained with the pure compounds’ standards. The sensory panel was performed under controlled environmental conditions (light 70–90 fc, temperature 22 ± 1 °C) in individual booths. For sensory evaluation, 5 µL of EOs were dissolved in 995 µL of distilled water; before the sensory evaluation, the mixture was thoroughly shaken. It should be highlighted that dissolving EOs in water is not optimal in terms of sensory evaluation. This option was used since one of the aims of our research was to compare the instrumental analysis approach (HS-SPME-GC-MS) and the human-involving approach; however, the HS-SPME technique is strongly sensitive to organic solvent presence—as a result of its low boiling points, the molecules of EtOH, MeOH, or other organic solvents would, with high efficiency, saturate the fiber and limit the actual extraction of VOCs. Therefore, to maintain the convergence of analytical approaches, water was used as a solvent for both instrumental and sensory analyses. During sensory analysis four tests were performed: (A) pairing the EOs with plant source material; (B) pairing the EOs with plant chemotype; (C) pairing the EOs with pure enantiomers standards of VOCs; and (D) pairing the plant material with pure enantiomeric standards of VOCs. This experimental design allowed us to check how representative the EOs were from the dried plants, the plant chemotype, and even pure enantiomer standards, and whether the use of instrumental analysis can fully replace the human senses.

### 2.7. Statistical Analysis

For statistical analysis, 13.3 Statistica software (StatSoft, Kraków, Poland) was used. For EOs yields and VOCs contribution statistical differences, one-way ANOVA was applied, including previous verification of normality and homogeneous variance by Levene’s test. For all relevant cases, standard deviation (**SD**) was applied. The hierarchical cluster analysis (**HCA**) with Ward’s linkage and Euclidean distance was applied and the strict (33%) Sneath’s criterium was used to highlight the sensory evaluation results. The analyses were made with a completely randomized design.

## 3. Results and Discussion

To investigate the relationship between plants’ VOCs enantiomeric distribution and its influence on the odor sensory quality of plants a comprehensive study with multiple approaches was performed. The quantity of EOs was determined by hydrodistillation with Deryng’s apparatus. Thereafter, the enantiomeric composition of EOs was determined with enantioselective GC-MS analysis. Parallelly, the enantioselective analysis of VOCs enantiomers emitted to plants’ headspace with the HS-SPME technique was performed. Finally, the sensory panel was carried out with the purpose to verify the ability of judges to match the EOs with the source plant material or pure VOCs enantiomers standards.

### 3.1. Essential Oil Yield

**Figure 1:**
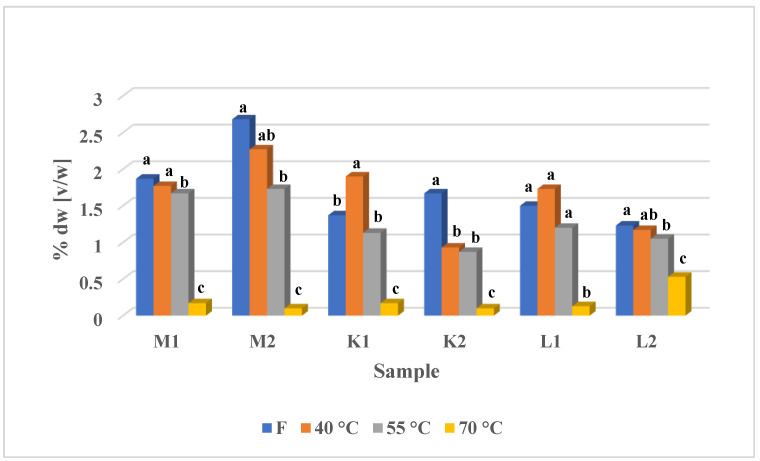
Essential oils yield: hydrodistillation resulted in various amounts of obtained EOs. Except K1 and K2 samples, the EOs yields did not differ significantly between F samples and those dried at 40 °C. For all samples, except K1 and L1, drying at 55 °C caused a significant decrease in EOs content, while drying at 70 °C for all samples was the one with the strongest EOs content decrease. Values followed with the same letters are not statistically different in Tukey’s test and one-way analysis of variance.

Hydrodistillation carried out with Deryng’s apparatus resulted in various amounts of obtained EOs (Figure 1). Except for K1 and K2 samples, the EOs yields did not differ significantly between F samples and those dried at 40 °C. For all samples, except K1 and L1, drying at 55 °C caused a significant decrease in EOs content, while drying at 70 °C for all samples was the most destructive. Such phenomena should relate to the sensitivity of plant tissue since higher temperatures possibly disintegrate the glandular trichomes structure. The trend of VOCs emission differentiation due to glandular trichomes disintegration was observed by Vallino et al. (2021) [13]. Additionally, the VOCs emission differentiation was confirmed by us in our earlier study [6]. The last to be discussed is the issue of EOs yielding considerable increase for K1 sample dried at 40 °C which was also reported before for coriander [14], laurel [15], and even mint [16], and may relate to the morphology of a particular mint variety or may be caused with partial degradation/polymerization of compounds during freeing process.

### 3.2. Enantioselective Analysis

All mint samples were analyzed by qualitative and quantitative, enantioselective (Cydex-B column) GC-MS with two approaches: EOs GC-MS analysis by liquid injection and VOCs emission pattern by HS-SPME (the examples of chromatograms may be found in Supplementary Materials File S1: TIC chromatograms). Table 2, Table 3, Table 4, Table 5, Table 6 and Table 7 present the identified VOCs enantiomers and their contribution to all samples (the details of identification results may be found in Supplementary Materials File S2: Table S1—Qualitative analysis of mint VOCs enantiomers). The most characteristic of mint compounds such as (−)-menthol, (+)-piperitone, (−)-menthone, and (+)-isomenthone was found with 100% of enantiomeric excess which agrees with earlier studies [17,18]; contrarily, (+)-menthyl acetate was found in the present study for mint K2 and L1. Moreover, the L1 mint sample, which was expected to be a linalool pathway mint, has presented a profile more characteristic of the carvone pathway. The reason for this issue may be linked to the cultivation conditions, which may be responsible for the plant metabolism change [1]. Unfortunately, it was impossible to separate carvone enantiomers with the applied capillary column since its stationary phase presented too low-resolution effectiveness [17].

**Figure 2:**
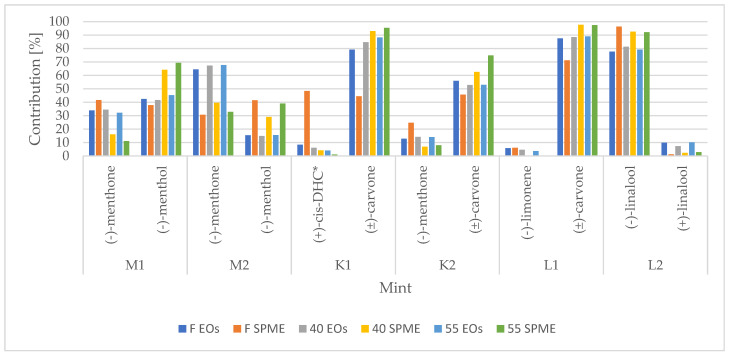
The comparison between EOs and SPME analysis illustrated in the example of most abundant VOCs enantiomers for each mint chemotype: even if the significant differences between major VOCs were observed, no clear patterns may be pointed out; * (+)-*cis*-dihydrocarvone; F EOs, 40 EOs, 55 Eos—refers to the GC-MS analysis of EOs by liquid injection; F SPME, 40 SPME, 55 SPME—refers to the analysis of source plant material by HS-SPME technique.

Significant differences in the distribution of VOC enantiomers (≥0.05%) were observed between EO and HS-SPME analysis in most cases, but no clear overall patterns were observed, as presented in Figure 2. Nevertheless, some interesting observations were made for samples within a particular mint chemotype. For menthol pathway samples, M1 (Table 2) and M2 (Table 3), during SPME analysis, the plant matrix significantly influenced the emission of monoterpenes enantiomers (both), namely α-pinene, sabinene, β-pinene, and limonene. Such observation may suggest that the plant tissue of the menthol pathway mint promotes the emission of molecules whose structure includes oxygen atoms. Contrarily, carvone pathway mint (Table 4 and Table 5) was not so predictable.

Plant tissue contains chiral compounds, such as carbohydrates present in cellulose and hemicellulose or some lignans, which may influence the distribution in mint headspace of particular VOCs enantiomers; nonetheless, no pattern was observed in general. Therefore, we suggest that the VOCs enantiomers emission is more linked with the changes in the plant tissue, forced by drying and/or other factors, which agrees with our earlier research [6]. Therefore, the enantiomeric distribution should not be applicable for sensory quality prediction of HSs in general, but the object must be investigated separately.

### 3.3. Sensory Analysis

**Figure 3:**
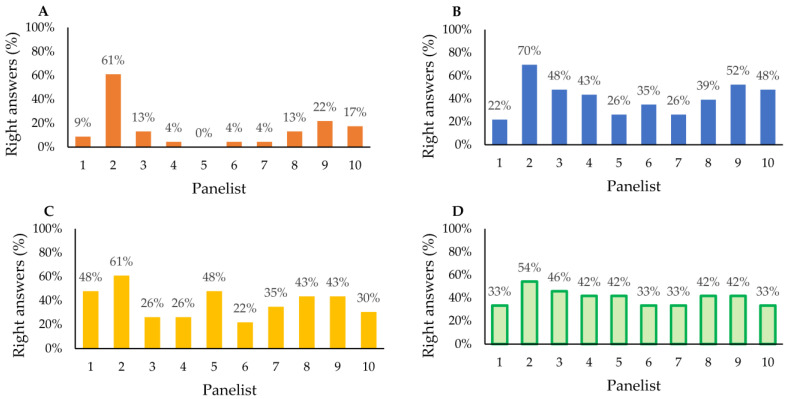
The effectiveness of pairing the samples during sensory panel: (**A**)—pairing EOs with source plants; (**B**)—pairing EOs with plant chemotypes; (**C**)—pairing EOs with pure enantiomers standards; (**D**)—pairing plant material with pure enantiomers standards. Ten panelists, with very poor results, were able to pair the EOs with their source plant materials (test A), while the results of the rest of the sensory tests for pairing the EOs with plant chemotype (test B), EOs with pure enantiomer standards (test C), and plant material with pure enantiomers standards (test D) gave a significantly better result.

**Figure 4:**
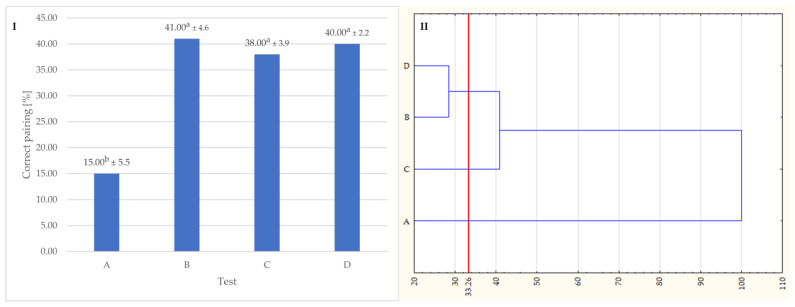
(**I**) Average results of pairing the samples during sensory tests; (**II**) HCA results for results of sensory tests. A—pairing EOs with source plants; B—pairing EOs with plant chemotypes; C—pairing EOs with pure enantiomers standards; D—pairing plant. The overall effectiveness of all pairing tests was lower than 50% (Figure 4I). Figure 4II presents the HCA results which show that sensory test B, C, and D effectiveness present the same homogeneous group, with accuracy oscillating at 40%, while test A effectiveness is significantly lower with an accuracy of 15%. Values followed by the same letters within a row are not statistically different in Tukey’s test and one-way analysis of variance (*p* < 0.05).

The aim of the sensory analysis was to investigate, in-depth, the relationships among enantiomeric VOCs emission—plant matter-sensory quality. Thus, four sensory tests have been applied to reveal that the chemistry of plants and EOs fragrance is a complex issue. Ten panelists, with very poor results, were able to pair the EOs with their source plant materials (test A). Meanwhile, the results of the rest of the sensory tests were significantly better, but still not optimal (Figure 3). The overall effectiveness of all pairing tests was lower than 50% (Figure 4I). Figure 4II presents the HCA results which show that sensory test B, C, and D effectiveness present the same homogeneous group, with accuracy oscillating at 40%, while test A effectiveness is significantly lower with an accuracy of 15%. Those results prove that the differences found during the instrumental approach in our previous research [6], have a reflection in human-involving approaches. Despite the significant differences in the samples’ volatile profile, the judges were not able to match the samples with sufficient effectiveness.

Observations similar to ours were reported by Kosakowska et al. (2019) [19] regarding the study with ‘Greek oregano’; even significant differences in EOs yield for oregano cultivated in different conditions, did not result in considerable differences during sensory analysis. Comparatively, the research conducted by Baczek et al. (2019) [20] with sweet basil resulted in opposite results—despite no considerable differences in EOs yield, the sensory analysis resulted in significant differences for part of notes, namely basilic, anisic, and spicy ones. In both cases, herbs have been recognized as ‘high-quality material’. Nevertheless, referred works of Kosakowska et al. (2019) [19], and Baczek et al. (2019) [20] aforementioned works, are rare regarding the evaluation of herbs aroma quality by applying the sensory evaluation approach. Contrarily, numerous scientific papers, found in prestigious journals, base the prediction of HSs quality on the EOs composition [14,21,22,23,24]. Asekun et al. (2006) [21] evaluated the quality of dried *M. longifolia* L. subsp. *Capensis* based on the reduction of potentially toxic pulegone content and advised to use for culinary purposes only and products with decreased amounts of pulegone, without any reflection on the sensory quality of the material. Similarly, Mohammed et al. (2020) [22] evaluated the rosemary EOs, referring to their antioxidant activity, and determined that the highest quality for meat processing are those with the best results during DPPH-method measurements, while no sensory quality was considered. There is no doubt that arguments raised by Asekun et al. (2006) [21] and Mohammed et al. (2020) [22] are important issues, however, they do not justify the categorical quality judgment.

The influence of particular VOCs enantiomers’ distribution on the aroma perception of food products intuitively is of great matter; thus, the differences between particular enantiomers’ smells have been clearly shown [25]. Nonetheless, in most cases of HSs, or obtained from EOs, the VOCs enantiomeric distribution is applied for authenticity or adulteration investigations, and not for sensory quality assessment [26,27,28,29]. This matter may be linked to the distribution of naturally volatile enantiomers, which seems to be characteristic for particular plant species, with stabilized enantiomeric excess which was perfectly shown for citrus EOs [27], goldenrod EOs [30], Madagascar plants EOs [31], and mint [18]. In terms of the present research, the most significant is VOCs enantiomeric distribution for mint, which according to Castillo et al. (2004) [18], despite the plant origin, in the majority of cases reach 100% of enantiomeric excess in favor of particular volatile enantiomer. Furthermore, as was proven in the current research, the plant tissue does not clearly affect the VOCs enantiomers emission, despite the presence of chiral compounds in the plant cell wall. That means that the VOCs enantiomers emission pattern is more related to the overall condition of plant tissue and may be variable due to applied post-harvest treatment, such as drying. Therefore, sensory quality should not be replaced with instrumental analysis, even if the sensory analysis is much more cost- and time-consuming. The reasons for that are multiple factors such as VOCs (including enantiomeric distribution) mutual ratio, and changes forced in plant tissue by post-harvest treatment.

## 4. Conclusions

The quality of herbs and spices is a complex issue that should be analyzed with respect to the purpose of the plant material. For odor sensory quality, the essential oils content in plant material is not crucial, but rather the distribution and emission of volatiles. The present study shows that despite the chiral character of *Mentha* spp. in plant tissue, the emission of volatile enantiomers is more dependent on the essential oil composition and the condition of the plant tissue than on its chiral composition. Therefore, the enantiomeric distribution of volatile organic compounds, even if used as the only chemical fingerprint for quality and adulteration measurements, should not be used as an odor quality marker. Moreover, the comparison of instrumental analysis (essential oils—GC-MS; source plants—HS-SPME-GC-MS) and the sensory panel are not interchangeable, since significant differences in the contribution of volatile enantiomers were not a factor that differentiated the odor quality of the panelists. The present study was performed based on the example of *Mentha* spp. plants, which differ in terms of essential oils composition and leaf morphological features. This may suggest that the trends may also be relevant for other plant species; however, further research on this issue should be considered.

## Figures and Tables

**Table 1:** Sample list and codes.

Cultivar and Chemotype	Type of Treatment	Sample Code
*Mentha × piperita* L. ‘Multimentha’ (menthol pathway)	frozen	M1_F
	dried at 40 °C	M1_40
	dried at 55 °C	M1_55
	dried at 70 °C	M1_70
*Mentha × piperita* L. ‘Swiss’ (menthol pathway)	frozen	M2_F
	dried at 40 °C	M2_40
	dried at 55 °C	M2_55
	dried at 70 °C	M2_70
*Mentha spicata* L. ‘Moroccan’ (carvone pathway)	frozen	K1_F
	dried at 40 °C	K1_40
	dried at 55 °C	K1_55
	dried at 70 °C	K1_70
*Mentha spicata* L. ‘Crispa’ (carvone pathway)	frozen	K2_F
	dried at 40 °C	K2_40
	dried at 55 °C	K2_55
	dried at 70 °C	K2_70
*Mentha × piperita* L. ‘Grapefruit’ (linalool pathway)	frozen	L1_F
	dried at 40 °C	L1_40
	dried at 55 °C	L1_55
	dried at 70 °C	L1_70
*Mentha × piperita* L. ‘Granada’ (linalool pathway)	frozen	L2_F
	dried at 40 °C	L2_40
	dried at 55 °C	L2_55
	dried at 70 °C	L2_70

**Table 2:** Contribution of volatile enantiomeric compounds in M1 mint EOs and headspace (HS-SPME).

Compound	ANOVA ^1^	[%]					
		F		40		55	
		EOs	SPME	EOs	SPME	EOs	SPME
α-(−)-pinene	*	0.45 ^a^	0.05 ^b^	0.54 ^a^	tr ^2^	0.49 ^a^	tr
α-(+)-pinene	*	0.33 ^a^	0.07 ^b^	0.58 ^a^	tr	0.53 ^a^	tr
(+)-sabinene	*	0.48 ^a^	0.18 ^b^	0.86 ^a^	0.09 ^b^	0.76 ^a^	0.08 ^b^
(−)-sabinene	*	0.16 ^a^	0.05 ^b^	0.28 ^a^	tr	0.24 ^a^	tr
β-(+)-pinene	*	0.54 ^a^	0.19 ^b^	0.87 ^a^	tr	0.81 ^a^	tr
β-(−)-pinene	*	0.52 ^a^	0.20 ^b^	0.89 ^a^	0.06 ^b^	0.83 ^a^	0.05 ^b^
(−)-limonene	*	4.69 ^a^	3.84 ^a^	5.88 ^a^	1.07 ^b^	5.33 ^a^	0.88 ^b^
(+)-limonene	*	0.16 ^b^	1.18 ^a^	0.18 ^b^	0.06 ^c^	0.18 ^b^	0.05 ^c^
(−)-linalool	*	0.26 ^b^	0.35 ^ab^	0.25 ^b^	0.40 ^a^	0.27 ^b^	0.39 ^a^
(−)-menthone	*	33.87 ^b^	41.56 ^a^	34.47 ^b^	16.10 ^c^	32.14 ^b^	11.10 ^d^
(+)-isomenthone	*	3.45 ^ab^	4.72 ^a^	3.00 ^ab^	2.29 ^b^	3.02 ^ab^	3.15 ^ab^
(+)-terpinen-4-ol	NS	0.20	0.18	0.16	0.25	0.22	0.28
(−)-terpinen-4-ol	NS	4.08	4.51	3.39	4.33	3.54	4.60
(−)-menthol	*	42.39 ^bc^	37.81 ^c^	41.56 ^bc^	64.14 ^a^	45.24 ^b^	69.28 ^a^
(−)-menthyl acetate	*	4.60 ^a^	1.18 ^b^	3.44 ^a^	3.11 ^a^	2.56 ^ab^	2.60 ^ab^
α-(+)-terpineol	NS	0.12	0.09	0.11	0.24	0.11	0.21
α-(−)-terpineol	NS	0.13	0.07	0.11	0.24	0.12	0.21
(+)-piperitone	*	3.40 ^b^	3.78 ^b^	3.27 ^b^	7.35 ^a^	3.47 ^b^	6.86 ^a^
(−)-*trans*-caryophyllene	NS	0.20	tr	0.15	0.14	0.13	0.13

Values followed by the same letters within a row are not statistically different in Tukey’s test and one-way analysis of variance. ^1^ NS—not statistically different; * Significant at *p* < 0.05; ^2^ tr—presence < 0.05%.

**Table 3:** Contribution of volatile enantiomeric compounds in M2 mint EOs and headspace (HS-SPME).

Compound	ANOVA ^1^	[%]					
		F		40		55	
		EOs	SPME	EOs	SPME	EOs	SPME
α-(−)-pinene	*	0.39 ^a^	tr ^3^	0.48 ^a^	0.10 ^b^	0.44 ^a^	0.05 ^b^
α-(+)-pinene	*	0.36 ^a^	0.10 ^b^	0.45 ^a^	0.08 ^b^	0.42 ^a^	0.05 ^b^
(+)-sabinene	*	0.52 ^a^	0.20 ^b^	0.66 ^a^	0.20 ^b^	0.52 ^a^	0.12 ^b^
(−)-sabinene	*	0.20 ^ab^	0.29 ^a^	0.25 ^a^	0.06 ^c^	0.19 ^b^	tr
β-(+)-pinene	*	0.60 ^a^	tr	0.72 ^a^	0.12 ^b^	0.67 ^a^	0.07 ^b^
β-(−)-pinene	*	0.50 ^a^	0.26 ^b^	0.62 ^a^	0.15 ^b^	0.57 ^a^	0.07 ^c^
(−)-limonene	*	0.86 ^a^	tr	0.44 ^b^	0.81 ^a^	0.39 ^b^	0.41 ^b^
(+)-limonene	*	0.06 ^b^	2.47 ^a^	tr	0.11 ^b^	tr	0.08 ^b^
(−)-linalool	*	0.39 ^c^	0.32 ^c^	0.38 ^c^	0.65 ^b^	0.40 ^c^	0.81 ^a^
(−)-menthone	*	64.40 ^a^	30.64 ^c^	67.28 ^a^	39.56 ^b^	67.74 ^a^	32.77 ^c^
(+)-isomenthone	*	5.14 ^a^	4.38 ^ab^	4.74 ^ab^	4.34 ^b^	5.08 ^ab^	5.07 ^ab^
(+)-terpinen-4-ol	*	0.43 ^b^	6.61 ^a^	0.25 ^b^	0.31 ^b^	0.36 ^b^	0.43 ^b^
(−)-terpinen-4-ol	*	4.10 ^b^	6.12 ^a^	1.82 ^d^	2.68 ^c^	1.75 ^d^	3.20 ^c^
(−)-menthol	*	15.43 ^c^	41.47 ^a^	14.87 ^c^	29.04 ^b^	15.55 ^c^	39.02 ^a^
(−)-menthyl acetate	NS	3.55	3.00	3.17	7.35	2.58	4.61
(±)-carvone ^2^	*	0.16 ^b^	0.43 ^b^	0.68 ^b^	6.19 ^a^	0.04 ^b^	4.02 ^a^
(+)-piperitone	*	2.49 ^c^	3.67 ^b^	2.76 ^c^	7.89 ^a^	2.87 ^c^	8.35 ^a^
(−)-*trans*-caryophyllene	*	0.42 ^b^	tr	0.42 ^b^	0.35 ^c^	0.39 ^bc^	0.76 ^a^

Values followed by the same letters within a row are not statistically different in Tukey’s test and one-way analysis of variance. ^1^ NS—not statistically different; * Significant at *p* < 0.05; ^2^ since carvone enantiomers were not separated it was not possible to clearly define if the samples contained enantiomers mixture or one enantiomer with 100% of enantiomeric excess; ^3^ tr—presence < 0.05%.

**Table 4:** Contribution of volatile enantiomeric compounds in K1 mint EOs and headspace (HS-SPME).

Compound	ANOVA ^1^	[%]					
		F		40		55	
		EOs	SPME	EOs	SPME	EOs	SPME
α-(−)-pinene	*	0.54 ^a^	tr ^3^	0.53 ^a^	0.05 ^b^	0.42 ^a^	0.05 ^b^
α-(+)-pinene	*	0.31 ^a^	tr	0.31 ^a^	0.03 ^b^	0.23 ^a^	tr
(+)-sabinene	*	0.49 ^a^	tr	0.41 ^a^	0.06 ^b^	0.34 ^a^	tr
(−)-sabinene	*	0.28 ^b^	2.62 ^a^	0.24 ^b^	tr	0.20 ^b^	tr
β-(+)-pinene	*	0.57 ^b^	0.71 ^a^	0.52 ^b^	0.05 ^d^	0.45 ^c^	0.05 ^d^
β-(−)-pinene	*	0.61 ^a^	tr	0.55 ^ab^	0.06 ^c^	0.48 ^b^	0.06 ^c^
(−)-limonene	*	6.79 ^a^	2.02 ^bc^	5.13 ^ab^	1.12 ^c^	4.10 ^ab^	0.45 ^c^
(−)-linalool	NS	0.31	tr	0.20	0.23	0.22	0.24
(−)-menthone	*	0.23 ^c^	tr	0.18 ^c^	0.70 ^b^	0.17 ^c^	1.48 ^a^
(+)-terpinen-4-ol	NS	0.12	0.16	0.05	tr	0.05	tr
(−)-terpinen-4-ol	NS	0.09	0.17	tr	0.05	tr	0.09
(+)-*cis*-dihydrocarvone	*	8.35 ^b^	48.38 ^a^	6.14 ^c^	4.24 ^c^	4.10 ^c^	1.13 ^d^
(−)-menthol	NS	0.14	tr	0.12	0.11	0.12	0.19
(+)-dihydrocarveol	*	1.72 ^b^	2.96 ^a^	0.41 ^b^	0.22 ^b^	0.30 ^b^	0.46 ^b^
(±)-carvone ^2^	*	79.11 ^c^	44.44 ^d^	84.73 ^bc^	92.88 ^a^	88.26 ^ab^	95.38 ^a^
(−)-dihydrocarveol	*	0.21 ^a^	tr	0.14 ^b^	tr	0.11 ^bc^	0.08 ^c^
(−)-*trans*-caryophyllene	*	0.16 ^d^	0.50 ^a^	0.30 ^c^	0.10 ^d^	0.41 ^b^	0.26 ^cd^

Values followed by the same letters within a row are not statistically different in Tukey’s test and one-way analysis of variance. ^1^ NS—not statistically different; * Significant at *p* < 0.05; ^2^ since carvone enantiomers were not separated it was not possible to clearly define if the samples contained enantiomers mixture or one enantiomer with 100% of enantiomeric excess; ^3^ tr—presence < 0.05%.

**Table 5:** Contribution of volatile enantiomeric compounds in K2 mint EOs and headspace (HS-SPME).

Compound	ANOVA ^1^	[%]					
		F		40		55	
		EOs	SPME	EOs	SPME	EOs	SPME
α-(−)-pinene	*	0.89 ^a^	tr ^3^	1.00 ^a^	0.08 ^b^	0.93 ^a^	0.07 ^b^
α-(+)-pinene	*	1.19 ^b^	tr	1.42 ^a^	0.10 ^c^	1.30 ^ab^	0.10 ^c^
(+)-sabinene	*	1.84 ^c^	3.48 ^a^	2.05 ^b^	0.25 ^d^	2.00 ^b^	0.21 ^d^
(−)-sabinene	*	0.48 ^bc^	3.52 ^a^	0.54 ^b^	0.05 ^c^	0.51 ^b^	0.05 ^c^
β-(+)-pinene	*	1.57 ^a^	tr	1.87 ^a^	0.14 ^b^	1.84 ^a^	0.12 ^b^
β-(−)-pinene	*	2.02 ^a^	tr	2.41 ^a^	0.20 ^b^	2.39 ^a^	0.19 ^b^
(−)-limonene	*	9.57 ^a^	tr	7.16 ^b^	1.44 ^c^	6.26 ^b^	0.95 ^cd^
(−)-linalool	*	1.29 ^b^	1.21 ^b^	1.45 ^ab^	0.97 ^c^	1.58 ^a^	1.27 ^b^
(−)-menthone	*	12.84 ^b^	24.67 ^a^	14.23 ^b^	6.89 ^c^	14.02 ^b^	7.94 ^c^
(+)-terpinen-4-ol	NS	0.37	0.27	0.24	0.18	0.25	0.18
(−)-terpinen-4-ol	*	1.55 ^a^	1.21 ^ab^	1.38 ^ab^	1.04 ^b^	1.42 ^ab^	1.21 ^ab^
(+)-*cis*-dihydrocarvone	*	0.19 ^b^	13.53 ^a^	0.15 ^b^	15.32 ^a^	0.22 ^b^	0.56 ^b^
(−)-*cis*-dihydrocarvone	NS	0.07	tr	0.15	tr	0.14	0.06
(−)-menthol	*	4.27 ^bc^	3.68 ^c^	4.72 ^abc^	5.53 ^ab^	5.08 ^abc^	5.82 ^a^
(−)-menthyl acetate	*	1.47 ^b^	0.62 ^b^	2.81 ^a^	0.75 ^b^	3.12 ^a^	1.30 ^b^
(+)-menthyl acetate	NS	0.45	tr	0.44	0.69	0.51	0.67
(+)-dihydrocarveol	NS	0.12	tr	0.11	tr	0.13	tr
(±)-carvone ^2^	*	55.90 ^c^	45.65 ^d^	52.83 ^c^	62.49 ^b^	52.98 ^c^	74.80 ^a^
(+)-piperitone	*	1.87 ^bc^	1.46 ^c^	2.09 ^bc^	2.89 ^a^	2.08 ^bc^	2.45 ^ab^
(−)-dihydrocarveol	*	0.81 ^ab^	0.31 ^b^	1.13 ^a^	0.49 ^b^	0.94 ^ab^	0.96 ^ab^
(−)-*trans*-caryophyllene	*	1.26 ^b^	0.39 ^c^	1.82 ^ab^	0.51 ^c^	2.28 ^a^	1.06 ^b^

Values followed by the same letters within a row are not statistically different in Tukey’s test and one-way analysis of variance. ^1^ NS—not statistically different; * Significant at *p* < 0.05; ^2^ since carvone enantiomers were not separated it was not possible to clearly define if the samples contained enantiomers mixture or one enantiomer with 100% of enantiomeric excess; ^3^ tr—presence < 0.05%.

**Table 6:** Contribution of volatile enantiomeric compounds in L1 mint EOs and headspace (HS-SPME).

Compound	ANOVA ^1^	[%]					
		F		40		55	
		EOs	SPME	EOs	SPME	EOs	SPME
α-(−)-pinene	NS	0.52	0.44	0.66	tr ^3^	0.62	tr
α-(+)-pinene	NS	0.39	0.32	0.48	tr	0.47	tr
(+)-sabinene	NS	0.73	0.71	0.83	tr	0.77	tr
(−)-sabinene	NS	0.23	0.22	0.27	tr	0.24	tr
β-(+)-pinene	NS	0.62	0.49	0.71	tr	0.70	tr
β-(−)-pinene	*	0.75 ^b^	0.50 ^c^	0.87 ^a^	tr	0.84 ^a^	0.06 ^d^
(−)-limonene	*	5.79 ^a^	6.11 ^a^	4.60 ^ab^	0.24 ^c^	3.63 ^b^	0.22 ^c^
(+)-terpinen-4-ol	*	0.27 ^a^	0.06 ^b^	0.09 ^b^	0.06 ^b^	0.09 ^b^	0.07 ^b^
(−)-terpinen-4-ol	NS	0.16	tr	0.07	0.08	0.06	0.07
(+)-*cis*-dihydrocarvone	*	0.74 ^b^	17.08 ^a^	1.09 ^b^	0.09 ^b^	1.26 ^b^	0.11 ^b^
(−)-menthol	NS	0.29	0.28	0.25	0.37	0.28	0.38
(−)-menthyl acetate	NS	0.36	0.44	0.37	0.27	0.36	0.38
(+)-menthyl acetate	NS	tr	tr	tr	0.29	tr	0.31
(+)-dihydrocarveol	*	0.67 ^a^	tr	0.26 ^c^	tr	0.53 ^b^	0.07 ^d^
(−)-borneol	*	0.78 ^b^	1.99 ^a^	0.72 ^bc^	0.57 ^c^	0.85 ^b^	0.52 ^c^
(±)-carvone ^2^	*	87.60 ^ab^	71.23 ^b^	88.53 ^ab^	97.70 ^a^	89.08 ^ab^	97.46 ^a^
(−)-*trans*-caryophyllene	NS	0.12	0.11	0.18	0.17	0.22	0.13

Values followed by the same letters within a row are not statistically different in Tukey’s test and one-way analysis of variance. ^1^ NS—not statistically different; * Significant at *p* < 0.05; ^2^ since carvone enantiomers were not separated it was not possible to clearly define if the samples contained enantiomers mixture or one enantiomer with 100% of enantiomeric excess; ^3^ tr—presence < 0.05%.

**Table 7:** Contribution of volatile enantiomeric compounds in L2 mint EOs and headspace (HS-SPME).

Compound	ANOVA ^1^	[%]					
		F		40		55	
		EOs	SPME	EOs	SPME	EOs	SPME
α-(−)-pinene	NS	0.05	tr ^2^	0.09	0.05	0.06	0.05
α-(+)-pinene	*	0.26 ^c^	0.08 ^d^	0.62 ^a^	0.07 ^d^	0.41 ^b^	0.11 ^d^
(+)-sabinene	*	0.40 ^bc^	0.25 ^d^	0.94 ^a^	0.28 ^cd^	0.65 ^b^	0.35 ^c^
(−)-sabinene	NS	0.05	tr	0.14	tr	0.09	0.06
β-(+)-pinene	*	0.35 ^c^	0.12 ^d^	0.76 ^a^	0.08 ^d^	0.55 ^b^	0.12 ^d^
β-(−)-pinene	*	0.45 ^c^	0.16 ^d^	0.99 ^a^	0.10 ^d^	0.70 ^b^	0.15 ^d^
(−)-limonene	*	0.21 ^b^	0.14 ^b^	0.16 ^b^	0.58 ^a^	0.13 ^b^	0.56 ^a^
(+)-limonene	NS	0.39	0.28	0.39	0.39	0.34	0.37
(−)-linalool	*	77.68 ^d^	96.31 ^a^	81.26 ^c^	92.53 ^ab^	79.22 ^cd^	92.16 ^b^
(+)-linalool	*	9.93 ^a^	1.26 ^c^	7.25 ^b^	2.38 ^c^	10.12 ^a^	2.81 ^c^
α-(+)-terpineol	NS	2.73	0.62	2.08	1.38	2.16	1.26
α-(−)-terpineol	*	7.50 ^a^	0.78 ^c^	5.31 ^b^	2.15 ^c^	5.57 ^b^	2.02 ^c^

Values followed by the same letters within a row are not statistically different in Tukey’s test and one-way analysis of variance. ^1^ NS—not statistically different; * Significant at *p* < 0.05; ^2^ tr—presence < 0.05%.

## Data Availability

Data are contained within the article or Supplementary Materials. Data are also available upon request.

## Supplementary Materials

The following supporting information can be downloaded at: https://www.mdpi.com/article/10.3390/foods12102057/s1, File S1: TIC chromatograms; File S2: Table S1—Qualitative analysis of mint VOCs enantiomers.

## References

[1] Başer K.H.C., Buchbauer G. (2016). Handbook of Essential Oils. Science, Technology, and Applications.

[2] Raghavan S. (2007). Handbook of Spices, Seasoning and Flavorings.

[3] Chua L.Y.W., Chong C.H., Chua B.L., Figiel A. (2019). Influence of Drying Methods on the Antibacterial, Antioxidant and Essential Oil Volatile Composition of Herbs: A Review. Food Bioprocess Technol..

[4] Thamkaew G., Sjöholm I., Galindo F.G. (2021). A Review of Drying Methods for Improving the Quality of Dried Herbs. Crit. Rev. Food Sci. Nutr..

[5] Díaz-Maroto M.C., Castillo N., Castro-Vázquez L., de Torres C., Pérez-Coello M.S. (2008). Authenticity Evaluation of Different Mints Based on Their Volatile Composition and Olfactory Profile. J. Essent. Oil Bear. Plants.

[6] Łyczko J., Kiełtyka-Dadasiewicz A., Skrzyński M., Klisiewicz K., Szumny A. (2023). Chemistry behind Quality—The Usability of Herbs and Spices Essential Oils Analysis in Light of Sensory Studies. Food Chem..

[7] Łyczko J., Masztalerz K., Lipan L., Lech K., Carbonell-Barrachina Á.A., Szumny A. (2020). Chemical Determinants of Dried Thai Basil (*O. Basilicum* Var. *Thyrsiflora*) Aroma Quality. Ind. Crops Prod..

[8] Łyczko J., Jałoszyński K., Surma M., García-Garví J.-M., Carbonell-Barrachina A.A., Szumny A. (2019). Determination of Various Drying Methods’ Impact on Odour Quality of True Lavender (*Lavandula Angustifolia* Mill.) Flowers. Molecules.

[9] Ludwiczuk A., Kiełtyka-Dadasiewicz A., Sawicki R., Golus J., Ginalska G. (2016). Essential Oils of Some Mentha Species and Cultivars, Their Chemistry and Bacteriostatic Activity. Nat. Prod. Commun..

[10] Alavez-Rosas D., Nguyen L.M.N., Keefover-Ring K. (2022). Retention Indices for Naturally-Occurring Chiral and Achiral Compounds on Common Gas Chromatography Chiral Stationary Phases. Results Chem..

[11] Meilgaard M.C., Civille G.V., Carr B.T. (2016). Sensory Evaluation Techniques.

[12] (2008). International Standard Organization Sensory Analysis—General Guidance for the Selection, Training and Monitoring of As-sessors—Part 1: Selected Assessors.

[13] Vallino M., Faccio A., Zeppa G., Dolci P., Cerutti E., Zaquini L., Faoro F., Balestrini R. (2022). Impact of Drying Temperature on Tissue Anatomy and Cellular Ultrastructure of Different Aromatic Plant Leaves. Plant Biosyst..

[14] Ghasemi Pirbalouti A., Salehi S., Craker L., Pirbalouti A.G., Salehi S., Craker L. (2017). Effect of Drying Methods on Qualitative and Quantitative Properties of Essential Oil from the Aerial Parts of Coriander. J. Appl. Res. Med. Aromat. Plants.

[15] Sellami I.H., Wannes W.A., Bettaieb I., Berrima S., Chahed T., Marzouk B., Limam F. (2010). Qualitative and Quantitative Changes in the Essential Oil of *Laurus nobilis* L. Leaves as Affected by Different Drying Methods. Food Chem..

[16] Ahmed A., Ayoub K., Chaima A.J., Hanaa L., Abdelaziz C. (2018). Effect of Drying Methods on Yield, Chemical Composition and Bioactivities of Essential Oil Obtained from Moroccan *Mentha pulegium* L.. Biocatal. Agric. Biotechnol..

[17] Chanotiya C.S., Pragadheesh V.S., Yadav A., Gupta P., Lal R.K. (2021). Cyclodextrin-Based Gas Chromatography and GC/MS Methods for Determination of Chiral Pair Constituents in Mint Essential Oils. J. Essent. Oil Res..

[18] Ruiz Del Castillo M.L., Blanch G.P., Herraiz M. (2004). Natural Variability of the Enantiomeric Composition of Bioactive Chiral Terpenes in *Mentha piperita*. J. Chromatogr. A.

[19] Kosakowska O., Węglarz Z., Bączek K. (2019). Yield and Quality of ‘Greek Oregano’ (*Origanum vulgare* L. subsp. *hirtum*) Herb from Organic Production System in Temperate Climate. Ind. Crops Prod..

[20] Baczek K., Kosakowska O., Gniewosz M., Gientka I., Weglarz Z. (2019). Sweet Basil (*Ocimum basilicum* L.) Productivity and Raw Material Quality from Organic Cultivation. Agronomy.

[21] Asekun O.T., Grierson D.S., Afolayan A.J. (2007). Effects of Drying Methods on the Quality and Quantity of the Essential Oil of *Mentha longifolia *L. subsp.* Capensis*. Food Chem..

[22] Mohammed H.A., Al-Omar M.S., Mohammed S.A.A., Aly M.S.A., Alsuqub A.N.A., Khan R.A. (2020). Drying Induced Impact on Composition and Oil Quality of Rosemary Herb, *Rosmarinus Officinalis* Linn. Molecules.

[23] Rohloff J., Dragland S., Mordal R., Iversen T.H. (2005). Effect of Harvest Time and Drying Method on Biomass Production, Essential Oil Yield, and Quality of Peppermint (*Mentha × piperita* L.). J. Agric. Food Chem..

[24] Rahimmalek M., Goli S.A.H. (2013). Evaluation of Six Drying Treatments with Respect to Essential Oil Yield, Composition and Color Characteristics of *Thymys Daenensis* Subsp. *Daenensis*. Celak Leaves. Ind. Crops Prod..

[25] Geithe C., Krautwurst D. (2015). Chirality Matters—Enantioselective Orthologous Odorant Receptors for Related Terpenoid Structures. Importance of Chirality to Flavor Compounds.

[26] Davies N.W., Larkman T., Marriott P.J., Khan I.A. (2016). Determination of Enantiomeric Distribution of Terpenes for Quality Assessment of Australian Tea Tree Oil. J. Agric. Food Chem..

[27] Bonaccorsi I., Sciarrone D., Cotroneo A., Mondello L., Dugo P., Dugo G. (2011). Enantiomeric Distribution of Key Volatile Components in Citrus Essential Oils. Rev. Bras. Farmacogn..

[28] Wang M., Zhao J., Avula B., Wang Y.H., Chittiboyina A.G., Parcher J.F., Khan I.A. (2015). Quality Evaluation of Terpinen-4-ol-Type Australian Tea Tree Oils and Commercial Products: An Integrated Approach Using Conventional and Chiral GC/MS Combined with Chemometrics. J. Agric. Food Chem..

[29] Wang M., Chittiboyina A.G., Parcher J.F., Ali Z., Ford P., Zhao J., Avula B., Wang Y.H., Khan I.A. (2019). *Piper Nigrum* Oil—Determination of Selected Terpenes for Quality Evaluation. Planta Med..

[30] Chanotiya C.S., Yadav A. (2008). Natural Variability in Enantiomeric Composition of Bioactive Chiral Terpenoids in the Essential Oil of *Solidago canadensis* L. from Uttarakhand, India. Nat. Prod. Commun..

[31] Moè Llenbeck S., Koè Nig T., Schreier P., Schwab W., Rajaonarivony J., Ranarivelo L. (1997). Chemical Composition and Analyses of Enantiomers of Essential Oils from Madagascar. Flavour Fragr. J..

