# Supplementary material for: Chemistry behind Quality—Emission of Volatile Enantiomers from Mentha spp. Plant Tissue in Relationship to Odor Sensory Quality
Source: Foods. 2023 May 19;12(10):2057. doi: 10.3390/foods12102057 (PMC10217043; doi:10.3390/foods12102057)
Supplement: Supplementary file 1 [file foods-12-02057-s001.zip › Supplementary materials File S1 TIC chromatograms.pdf]

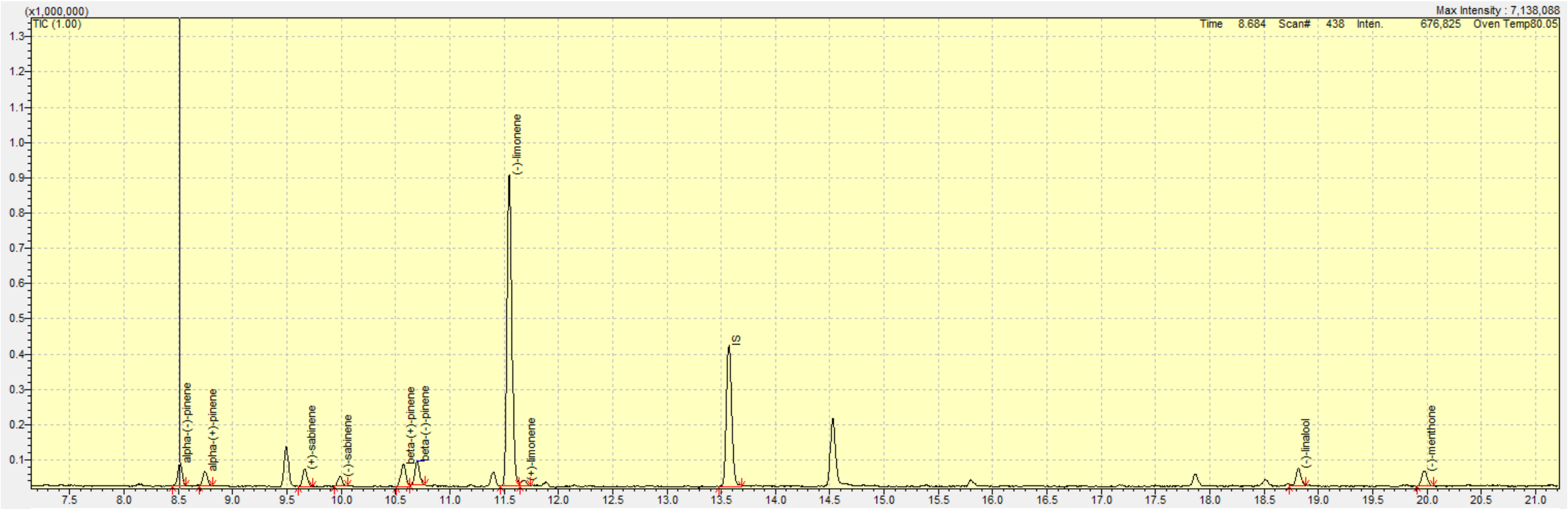

Figure S1 TIC chromatogram of K1 mint sample - part I

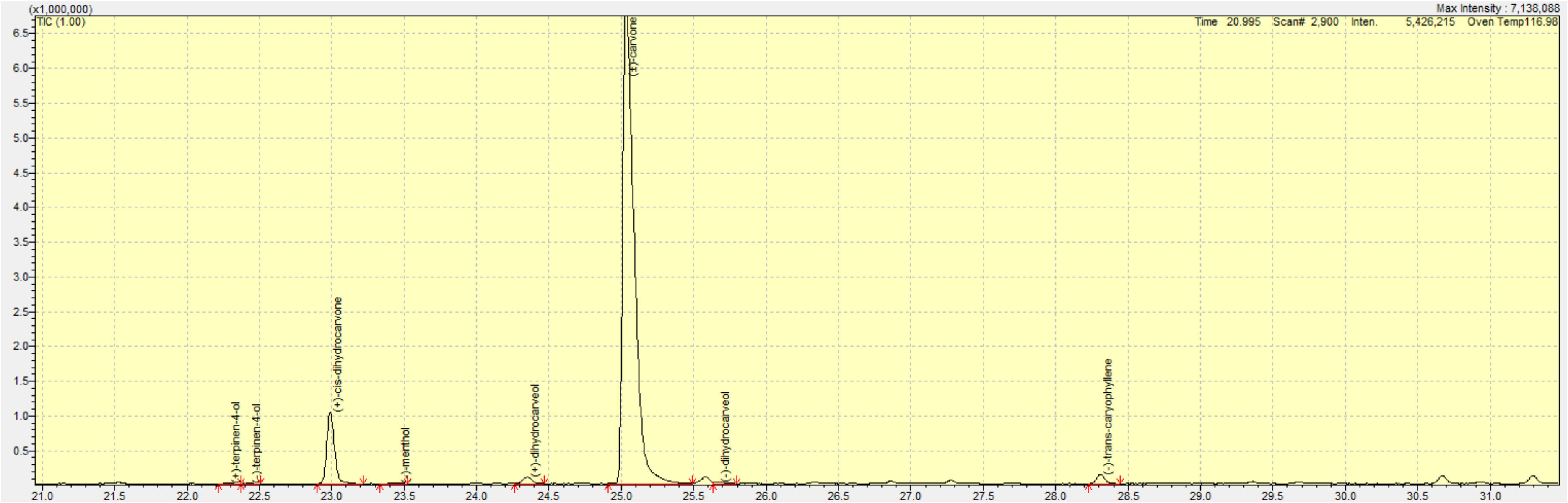

Figure S2 TIC chromatogram of K1 mint sample - part II

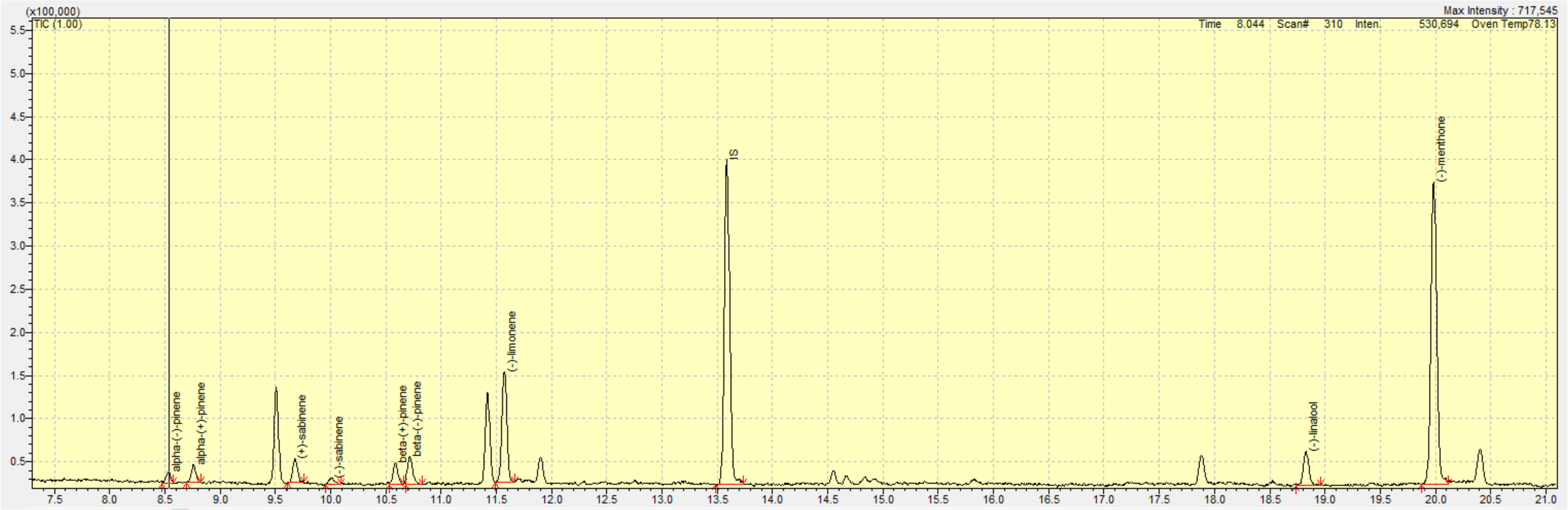

Figure S3 TIC chromatogram of K2 mint sample - part I

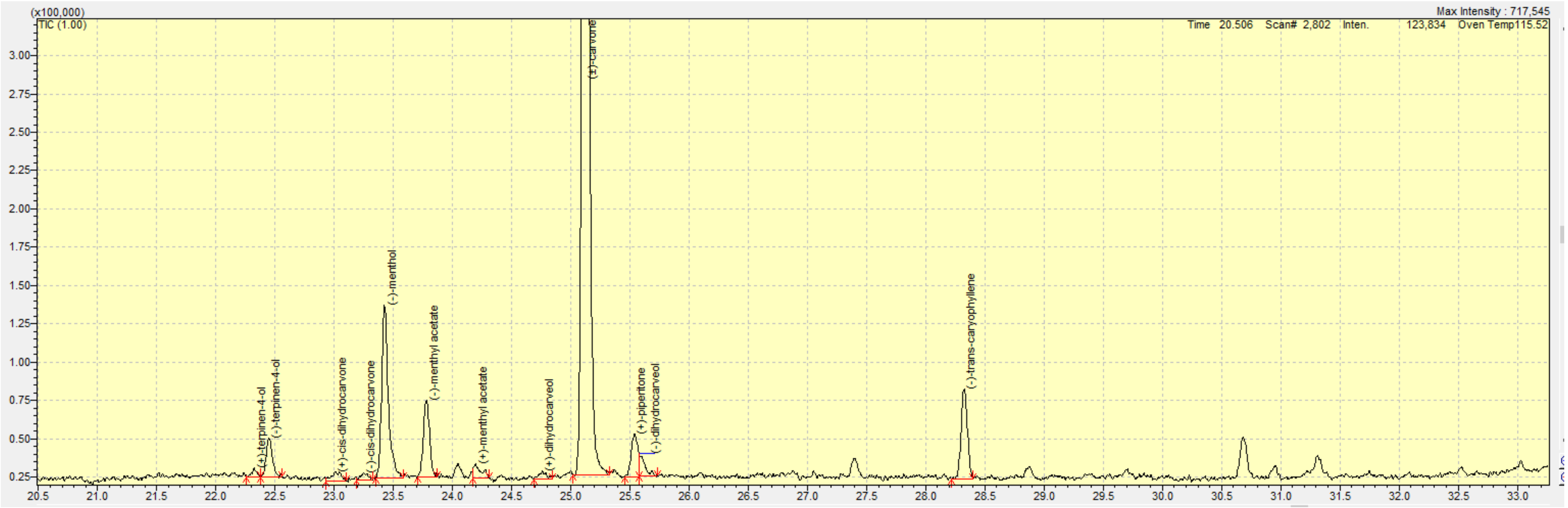

Figure S4 TIC chromatogram of K2 mint sample - part II

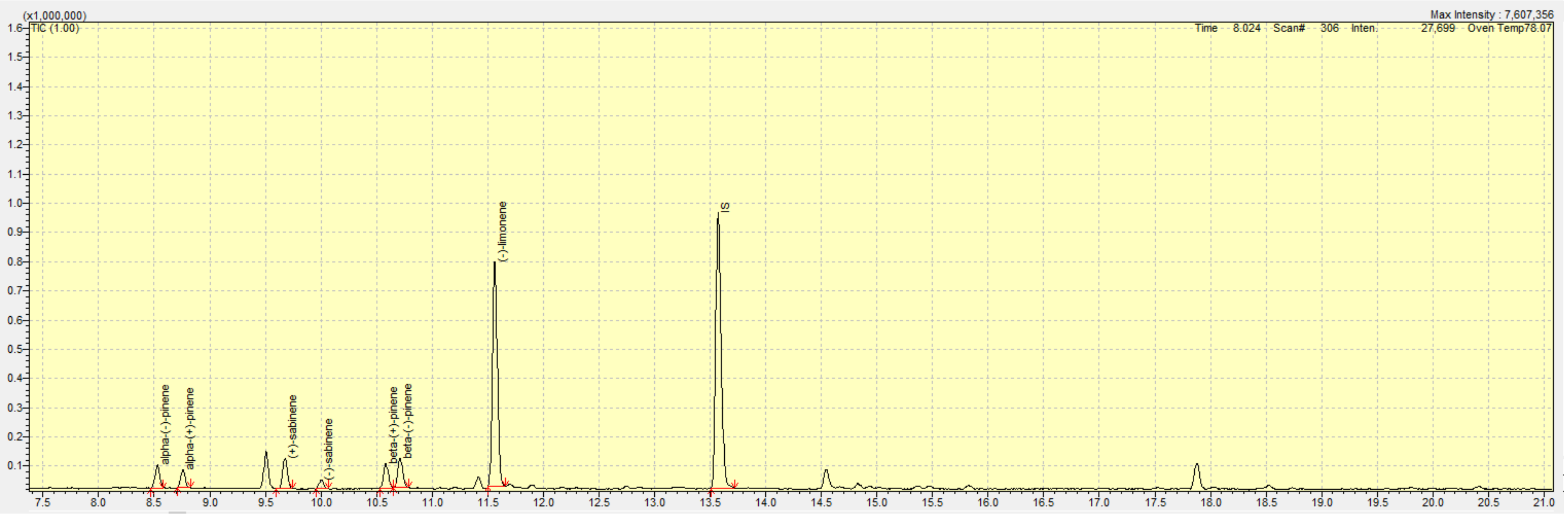

Figure S5 TIC chromatogram of L1 mint sample - part I

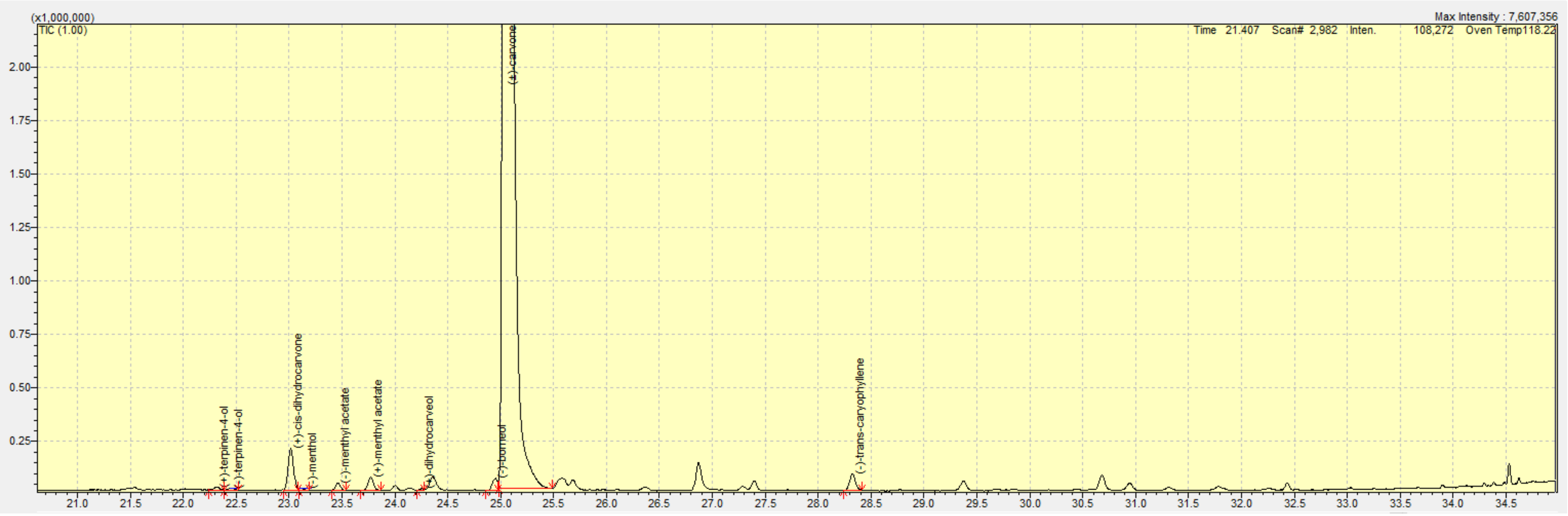

Figure S6 TIC chromatogram of L1 mint sample - part II

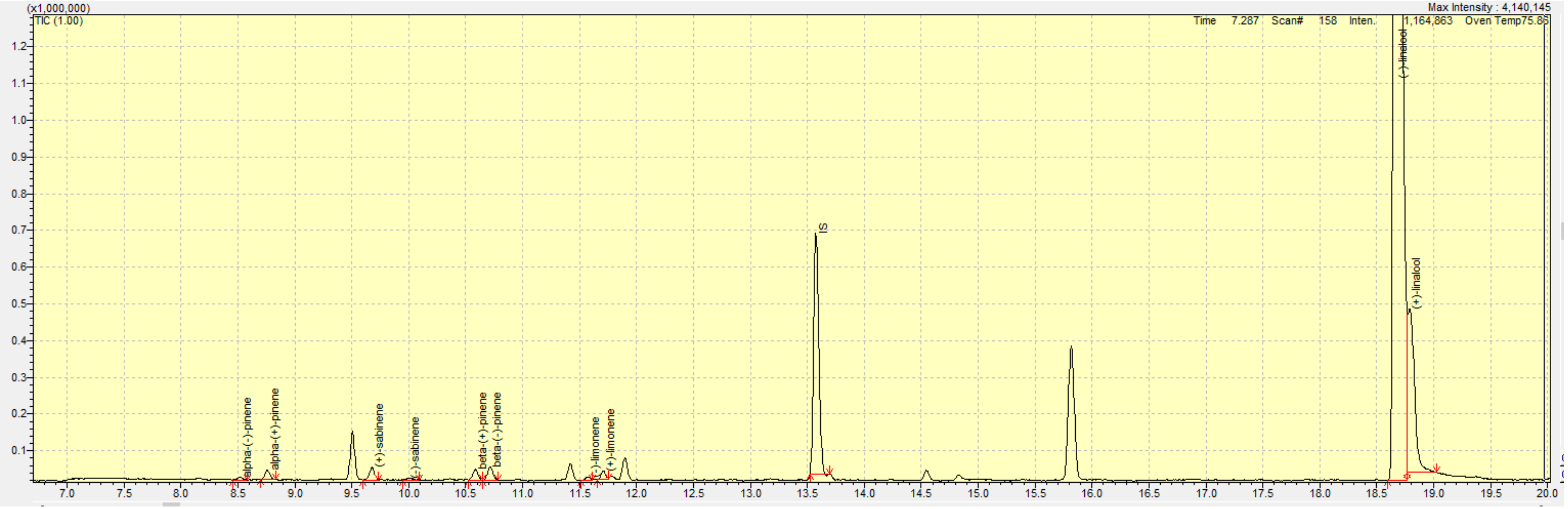

Figure S7 TIC chromatogram of L2 mint sample - part I

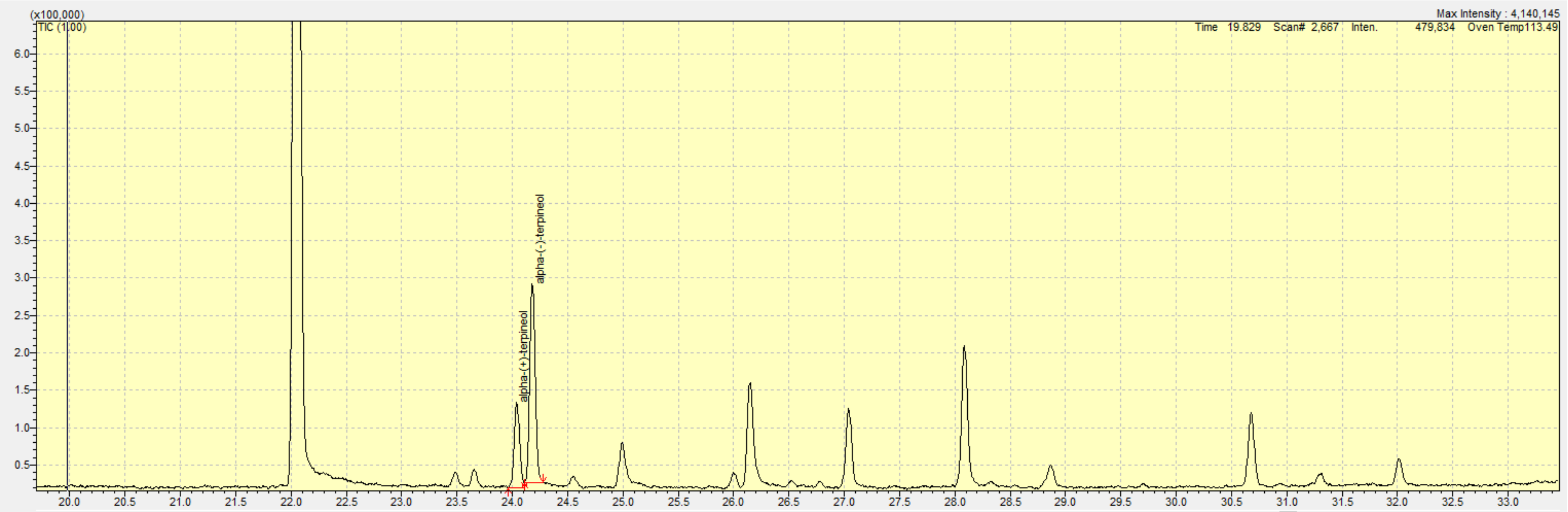

Figure S8 TIC chromatogram of L2 mint sample - part II

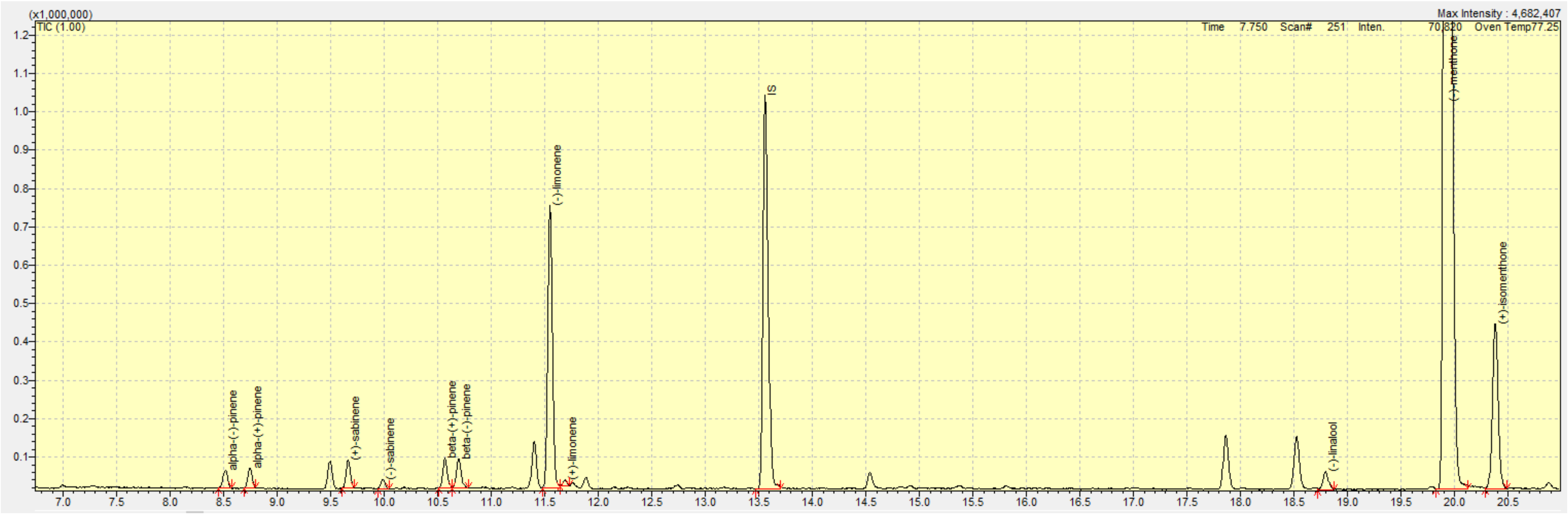

Figure S9 TIC chromatogram of M1 mint sample - part I

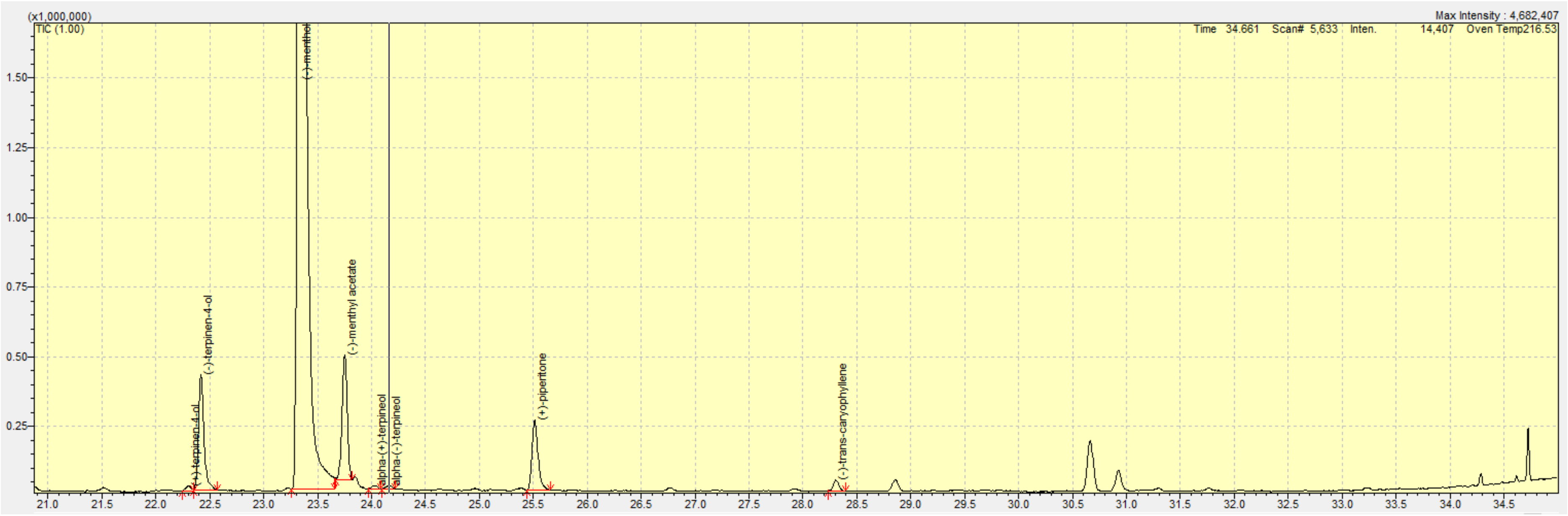

Figure S10 TIC chromatogram of M1 mint sample - part II

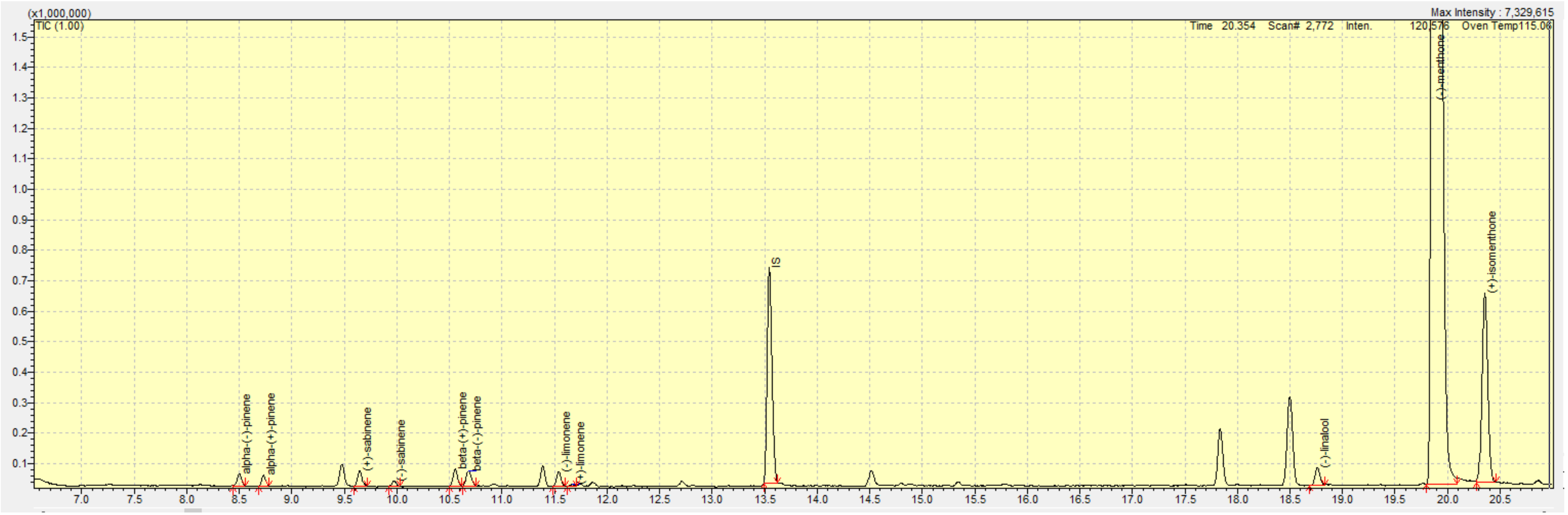

Figure S11 TIC chromatogram of M2 mint sample - part I

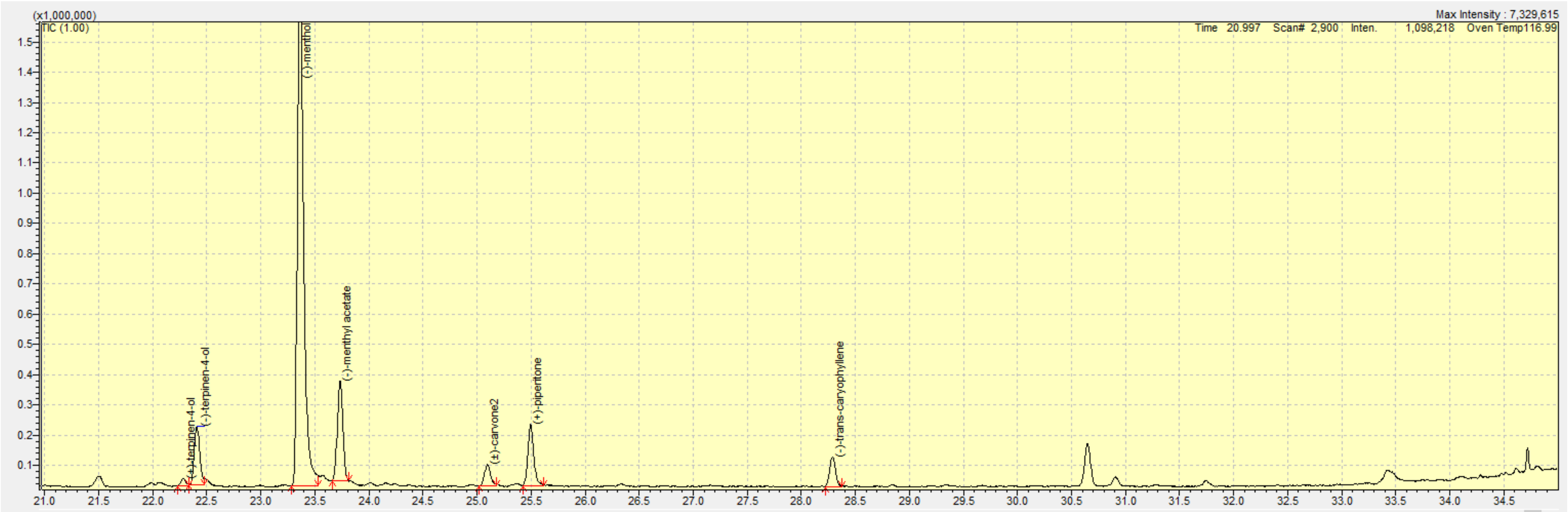

Figure S12 TIC chromatogram of M2 mint sample - part II
